# Urinary 2-Hydroxyglutarate Enantiomers Are Markedly Elevated in a Murine Model of Type 2 Diabetic Kidney Disease

**DOI:** 10.3390/metabo11080469

**Published:** 2021-07-21

**Authors:** Judy Baek, Subramaniam Pennathur

**Affiliations:** 1 Department of Molecular and Integrative Physiology, University of Michigan, Ann Arbor, MI 48105, USA; judybaek@umich.edu; 2 Division of Nephrology, Department of Internal Medicine, University of Michigan, Ann Arbor, MI 48105, USA

**Keywords:** diabetic kidney disease, biomarker, mitochondria, 2-hydroxyglutarate, TCA cycle

## Abstract

Metabolic reprogramming is a hallmark of diabetic kidney disease (DKD); nutrient overload leads to increased production of metabolic byproducts that may become toxic at high levels. One metabolic byproduct may be 2-hydroxyglutarate (2-HG), a metabolite with many regulatory functions that exists in both enantiomeric forms physiologically. We quantitatively determined the levels of L and D-2HG enantiomers in the urine, plasma, and kidney cortex of *db*/*db* mice, a pathophysiologically relevant murine model of type 2 diabetes and DKD. We found increased fractional excretion of both L and D-2HG enantiomers, suggesting increased tubular secretion and/or production of the two metabolites in DKD. Quantitation of TCA cycle metabolites in *db*/*db* cortex suggests that TCA cycle overload and an increase in 2-HG precursor substrate, α-ketoglutarate, drive the increased L and D-2HG production in DKD. In conclusion, we demonstrated increased 2-HG enantiomer production and urinary excretion in murine type 2 DKD, which may contribute to metabolic reprogramming and progression of diabetic kidney disease.

## 1. Introduction

Diabetic kidney disease (DKD) is the most prevalent etiology of end-stage kidney disease (ESKD), accounting for ~40% of all ESKD cases in the United States [1] and worldwide. DKD is marked by dramatic metabolic reprogramming, in particular of proximal tubules, due to hypoxia from microvascular damage, increased energy demand due to the increased glucose load, mitochondrial dysfunction, and excess nutrient burden [2]. Under normal physiologic conditions, proximal tubules prefer fatty acids and glutamine as their main energy sources [3], but in diabetes, proximal tubules increase glucose utilization. As a result, tubules upregulate glucose flux into glycolysis [4] and increase lactate production and excretion in the urine [5,6]. In addition, increased glucose flux into the TCA cycle [4], de novo lipogenic pathways [7,8,9,10,11], and secondary glycolytic pathways [12] are thought to upregulate the production of toxic metabolic byproducts that in turn contribute to DKD pathogenesis in human and model system studies.

One potential toxic metabolic byproduct may be 2-hydroxyglutarate (2-HG), which is derived from α-ketoglutarate by the promiscuous catalysis of various metabolic enzymes. 2-HG exists as two enantiomers, D-2HG and L-2HG. Of note, these enantiomers are normally produced and excreted in urine but also converted back to α-ketoglutarate by the actions of mitochondrial enzymes D-2HG dehydrogenase (D-2HGDH) and L-2HG dehydrogenase (L-2HGDH) [13]. D-2HG is most well-known as an oncometabolite that accumulates due to active site mutations in isocitrate dehydrogenase (IDH) 1 and IDH 2 that generate D-2HG [14,15]. D-2HG, however, can also be generated under physiological conditions by the action of phosphoglycerate dehydrogenase (PHGDH), although high production may only be achieved with PHGDH copy number amplification [16]. L-2HG is thought to be mainly generated under hypoxic and acidic conditions by the promiscuous action of malate and lactate dehydrogenase (MDH and LDH refs.: [17,18,19]).

Both enantiomers are structural analogs of α-ketoglutarate and can therefore compete for proteins and enzymes that use α-ketoglutarate as cofactor or substrate, such as α-ketoglutarate-dependent dioxygenases [20]. Apart from its role in oncogenesis, studies have found a physiological role of 2-HG in regulating T cell fate and function, in which accumulation of L-2HG precedes T cell differentiation and alters both T cell metabolism and epigenetics [21,22]. A recent study found decreased production of L and D-2HG in a murine model of lipopolysaccharide-induced endotoxemia, driven by increases in D-2HGDH and L-2HGDH protein levels [23]. In conclusion, 2-HG is a metabolite with physiological regulatory roles, and alterations in its levels can lead to significant changes in cellular function.

Elevations in L-2HG are particularly of interest because its production occurs in hypoxia and acidic conditions and, therefore, can serve as a marker of metabolic reprogramming in DKD. We hypothesized that in DKD, due to increased lactate production and glucose-flux into the TCA cycle with potential consequential accumulation of α-ketoglutarate, would result in increased 2-HG production, in particular of L-2HG. We investigated the level of D and L-2HG in urine, plasma, and kidney cortex of B6.BKS(D)-Lepr db/J (*db*/*db*) mice, which is a pathophysiologically relevant model of type 2 diabetes DKD [24].

## 2. Results

### 2.1. Urine, Plasma Concentration, and Percent Fractional Excretion of D and L-2HG

**Figure 1:**
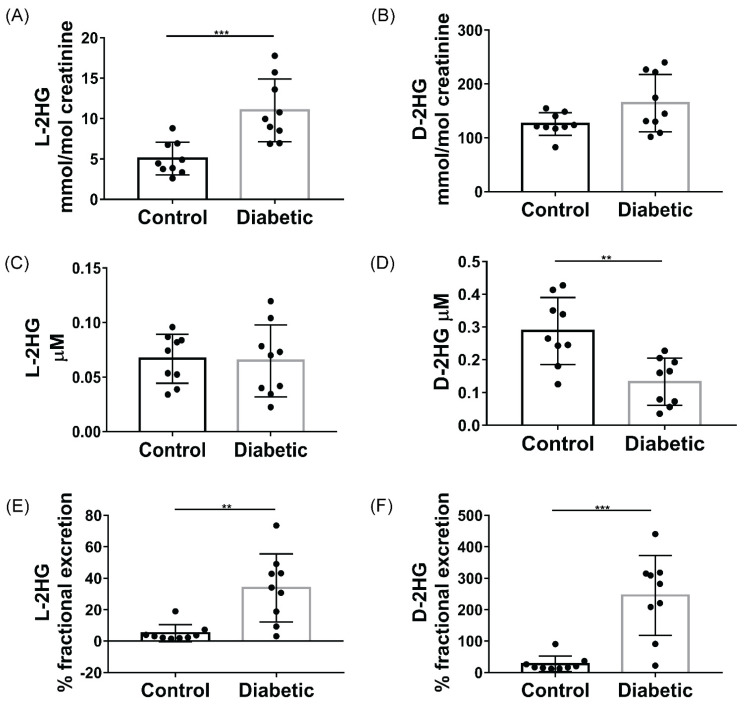
Concentration of L and D-2HG (±SD) in plasma and urine and calculated percent fractional excretion of each metabolite from Control (*db*/*+*) and Diabetic (*db*/*db*) and mice. (**A**) Urinary L-2HG and (**B**) Urinary D-2HG levels normalized creatinine, (**C**) plasma L-2HG levels, (**D**) plasma D-2HG levels, (**E**) % fractional excretion of L-2HG, (**F**) % fractional excretion of D-2HG. Student’s *t*-test, ** = *p*-value ≤ 0.001, *** = *p*-value ≤ 0.0001.

**Figure 2:**
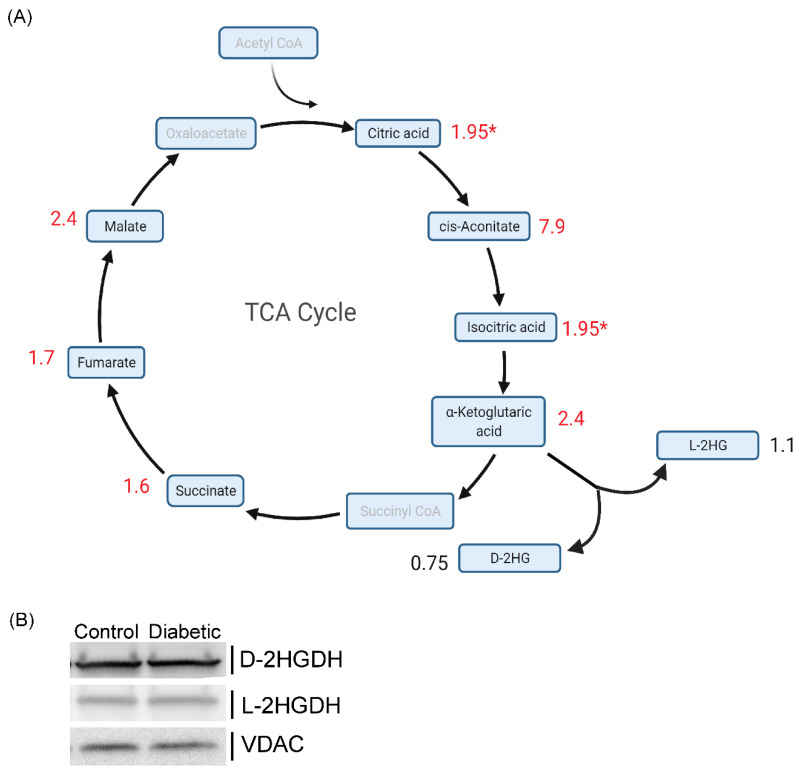
Kidney cortex levels of TCA cycle metabolites drive D and L-2HG production. (**A**) Schematic of TCA cycle with fold change (Control *db*/*+* vs. diabetic *db*/*db*) denoted (red if *p* ≤ 0.05 and black if did not meet significance). Metabolites in gray were not measured in the study. *: Citrate and isocitrate were measured as one transition. (**B**) Western blot of *db*/*+* and *db*/*db* cortex for D-2HGDH, L-2HGDH, and VDAC (*n* = 4). Figure 2A was created with BioRender.com.

Derivatization of 2-HG with chiral agent (+)-O,O′-Diacetyl-L-tartaric anhydride (DATAN) allows for the separation of the enantiomers on a reverse-phase column. Using this method, we quantitatively measured the levels of D and L-2HG from diabetic *db*/*db* and control *db*/*+* littermates. We found increased levels of L-2HG in the *db*/*db* urine (Figure 1A), while the plasma levels were not different between the groups (Figure 1C). Of note, measured metabolites in the urine were normalized to urinary creatinine concentration, which is an accepted method of determination for urinary protein or metabolite levels in DKD. The major disadvantage of spot urine samples is that the hydration status and the resultant rate of urine production make urine concentration variable. In addition, disease-specific differences in concentrating function and osmotic diuresis in DKD can result in substantial variations in metabolite concentrations. In an attempt to adjust for this variation, urinary creatinine (UCr) concentration is most commonly used in a ratio to normalize analyte quantification for specimen concentration [25]. D-2HG levels were elevated in the *db*/*db* urine (Figure 1B), although the levels did not reach significance (*p* = 0.056), while the plasma levels in the *db*/*db* urine were significantly decreased (Figure 1D). Increased urinary excretion of metabolites can be due to increased filtration or increased production by the kidney. Hyperfiltration occurs in *db*/*db* mice and remains elevated compared to controls until at least 28 weeks of age [26], although the glomerular filtration rate eventually declines with age [5,26]. The reduction in plasma D-2HG levels in *db*/*db* mice, in particular, suggests that the increased glomerular filtration rate (GFR) may lead to increased urinary excretion of D-2HG. We then calculated the fractional excretion of D (Figure 1F) and L-2HG (Figure 1E), which was calculated with the formula: [D or L-2HG]_Urine_ × [Creatinine]_Plasma_/[D or L-2HG]_Plasma_ × [Creatinine]_Urine_. Fractional excretion of both D and L-2HG was significantly elevated in the *db*/*db* mice, suggesting that the increased urinary levels of both metabolites are independent of GFR and systematic production of the metabolite, and is likely due to increased tubular excretion, decreased tubular reabsorption, or increased kidney production of D and L-2HG.

### 2.2. Elevated TCA Cycle Metabolites Drive Increased D and L-2HG Fractional Excretion

In order to assess for potential factors leading to increased D and L-2HG urinary levels, we quantified the levels of TCA cycle metabolites and D and L-2HG levels in the *db*/*db* and *db*/*+* mice kidney cortex. We found significant elevations in all TCA cycle metabolites (Figure 2A and Table 1), including α-ketoglutarate, the substrate for D and L-2HG. In our previous work (Ref. [4]), α-ketoglutarate was not reliably detected; hence, quantification was not feasible. Our new method increased sensitivity due to improvements in hydrophilic interaction chromatography; improvements in sensitivity and peak shapes of organic acids have been observed with the addition of phosphate [27] or phosphate-containing additives [28] to the samples or mobile phases. Phosphates are thought to shield metal ion interaction with organic acids, improving peak shape and sensitivity. Kidney cortical levels of D and L-2HG were not significantly different between *db*/*db* and *db*/*+* (Figure 2A), potentially due to the excretion of metabolites into the urine, maintaining normal intracellular levels of D and L-2HG. In order to determine whether levels are in D and L-2HG levels are due to changes in D-2HGDH and L-2HGDH protein levels, we assessed the levels of the enzymes with Western blots (Figure 2B) and found no difference between the two groups. We measured TCA cycle metabolites from the plasma and urine of *db*/*db* and *db*/*+* mice (Supplementary Materials Tables S1 and S2, respectively). There was no significant difference between the two groups in the metabolite levels in either plasma or urine. Only α-ketoglutaric acid levels in urine samples met *p*-value < 0.05, but failed to meet significance with multiple testing corrections. The urine and plasma data suggest that elevations in 2-HG are localized to the kidney.

### 2.3. Hyperglycemia Increases D and L-2HG Production in Human Proximal Tubular HK-2 Cells

**Figure 3:**
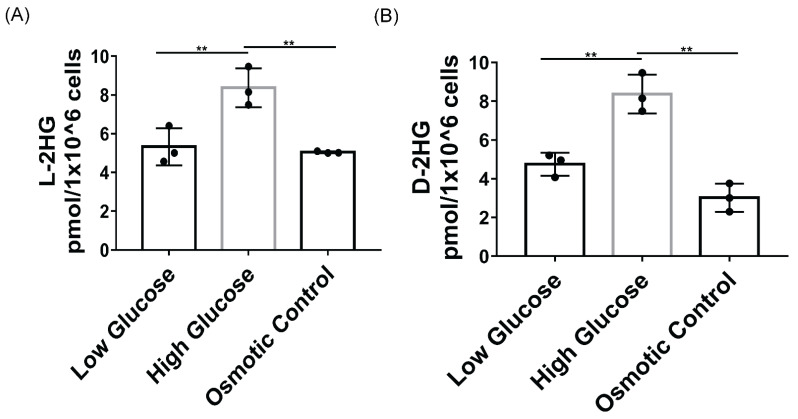
HK-2 production of D and L-2HG in high glucose media. (**A**) L-2HG and (**B**) 2-HG levels from HK-2 cells cultured in 5 mM glucose (low glucose), 25 mM glucose (high glucose), 5 mM glucose with 20 mM mannitol (osmotic control). One-way ANOVA with Tukey’s post hoc correction, ** = *p*-value ≤ 0.01.

As a proof-of-concept, we cultured human proximal tubular HK-2 cells in low glucose (5 mM), high glucose (25 mM), and low glucose (5 mM) with mannitol (20 mM) as osmotic control for 48 h and assessed the levels of D and L-2HG production (Figure 3A,B). Studies have found that HK-2 cell exposure to hyperglycemic conditions leads to metabolic reprogramming and increased glucose metabolism and lactate production [29]. We found that in hyperglycemic conditions, cellular levels of D and L-2HG increased compared to low glucose and osmotic control conditions, suggesting that a diabetic milieu can lead to increased D and L-2HG production. LC/MS measurements of TCA cycle metabolites from HK-2 cells in low glucose, high glucose, and low glucose with osmotic control are displayed in Supplementary Materials Table S3. Our analysis showed that all TCA cycle metabolites were elevated under the high glucose conditions above both the low glucose and osmotic control group. We did not detect 2-HG in the media consistently (below the range of detection in several samples, data not shown). These data are consistent with mouse kidney cortex data for TCA cycle intermediates. While 2-HG in media was not analogous to in vivo mouse data, this is likely due to the fact that transformed cell culture models are not fully representative of normal in vivo tissue. From our findings, we propose that increased glucose utilization in DKD can lead to increased levels of TCA cycle metabolites, including α-ketoglutarate and drive 2-HG production.

## 3. Discussion

In summary, we found elevated levels of D and L-2HG enantiomers in *db*/*db* urine. Increased fractional excretion of D and L-2HG in *db*/*db* mice suggests that increased secretion or kidney production of D and L-2HG underline elevated levels of D and L-2HG in the DKD model. To our knowledge, this is the first investigation of 2-HG enantiomers in a murine model of DKD. A recent study by Hyeon et al. found that 2-HG was one of 16 urine metabolites in type-1 diabetic streptozotocin mice whose levels were restored to baseline by treatment with the RAS inhibitor losartan [30]. This finding is consistent with our finding that overall levels of 2-HG are increased in the urine of *db*/*db* mice and suggests that it may serve as a biomarker for DKD. Cheng et al. [31] examined D and L-2HG urinary levels in diabetic and non-diabetic patients but found no significant differences in the levels between the two groups for both enantiomers, potentially because of the relatively small sample size and because elevations of 2-HG may only occur in the context of DKD, and not necessarily in diabetes.

2-HG has been demonstrated in several circumstances to negatively regulate mitochondrial metabolism. In the context of IDH mutations, D-2HG has been found to inhibit ATP synthase activity [32] and impair α-ketoglutarate dehydrogenase in cardiac muscles and decrease mitochondrial membrane potential [33]. A study of L-2HGDH global knockout (KO) mice found the highest tissue accumulation of L-2HG in the kidney of the KO mice, compared to other metabolically active organs, liver, and muscles. In the absence of L-2HGDH, the kidney displayed a reduction in TCA cycle metabolites, proposed to occur due to inhibition of α-ketoglutarate dehydrogenase activity by L-2HG [34]. α-ketoglutarate dehydrogenase generates NADH, serves as a redox sensor for the mitochondria, and regulates mitochondrial energy production [35].

In our studies, the downstream TCA intermediates distal to α-ketoglutaric acid are elevated in addition to 2 HG, suggesting that not all α-ketoglutaric acid is converted to 2-HG. In addition, as the TCA cycle serves as a hub for central carbon metabolism, it has many sources of input into the cycle, and therefore downstream metabolite levels could be changed even if substantial α-ketoglutaric acid is converted to 2-HG. While our data demonstrated significant elevations of TCA cycle metabolites in the *db*/*db* mice, multiple metabolomics studies of DKD patient urine have found disparate regulation of different TCA cycle metabolites. Studies have shown that aconitic acid and citrate/isocitrate urinary levels are downregulated in DKD patients in comparison to diabetic patients [36,37,38] and potentially predict DKD progression [39], whereas urinary fumarate and malate levels are upregulated in DKD [36]. Since the *db*/*db* mouse is an early model of DKD, clinical findings are likely to represent disease states more advanced than those represented by the mouse model, and it is likely that the TCA cycle becomes progressively more dysregulated with disease progression. We propose that 2-HG may play a role in mediating metabolic dysfunction in DKD.

Although tissue levels of D and L-2HG were not elevated and were instead reflected in the urine in the *db*/*db* mice, in the context of falling GFR and tubular damage, 2-HG levels may become elevated and become a uremic solute. The kidneys, in particular the tubules, maintain metabolic homeostasis by excreting excess metabolic intermediates into the urine, such as short-chain acylcarnitines [40]. 2-HG may be similarly excreted to reduce metabolic stress. A limitation of our study is that while the cortex is mainly composed of proximal tubules by volume, it still represents a mixture of different cell types in the kidney, and the spatial localization of 2-HG remains unknown. Mass spectrometry imaging (MSI) with desorption electrospray ionization mass spectrometry (DESI) and matrix-assisted laser desorption/ionization (MALDI)-time of flight (TOF) have been used in human and murine models of DKD for compartment-specific expression of metabolites [41,42,43], and 2-HG detection with MALDI-TOF has been accomplished in brain tumors with IDH mutations [44]. However, no current MALDI-TOF data are available for 2-HG levels in DKD. To understand whether alterations in the TCA cycle and 2-HG are specific to proximal tubules in DKD, a future MSI imaging study may provide compartment-specific spatial information.

Elevations in 2-HG may also lead to altered epigenetic programming, in particular by inhibiting α-ketoglutarate-dependent dioxygenases. Altered methylation patterns have been associated with DKD in the Chronic Renal Insufficiency Cohort (CRIC) [39], Pima Indians [45], and the Diabetes Control and Complications Trial/Epidemiology of Diabetes Interventions and Complications cohort (DCCT/EDIC) [46]. Future studies of clinical samples from these cohorts may uncover the role of 2-HG in epigenetic regulation in DKD.

## 4. Materials and Methods

Materials: Male BKS *db*/*db* mice (BKS.Cg-m +/+ Lepr db/J) and littermate controls (*db*/*+*) were purchased from Jackson Labs (Bar Harbor, ME, USA) at 12 wks of age. L-α-Hydroxyglutaric acid disodium salt (L-2HG), D-α-Hydroxyglutaric acid disodium salt (D-2HG), L-α-Hydroxyglutaric acid-_13_C^5^ disodium salt, α-ketoglutarate, α-ketoglutarate, cis-aconitate, citrate, fumarate, malate, succinate, glucose, mannitol, ammonium formate, formic acid, (+)-O,O′-Diacetyl-L-tartaric anhydride (DATAN), and dichloromethane were purchased from Sigma-Aldrich, Miamisburg, OH, USA. _13_C^5^ α-ketoglutarate, _13_C^6^ citrate, _13_C^4^ fumarate, _13_C^4^ malate, _13_C^4^ succinate were purchased from Cambridge Isotope Labs Inc., Tewksbury, MA, USA. Human kidney 2 (HK2) cells were purchased from American Type Culture Collection (ATCC^®^ CRL-2190™, Manassas, VA, USA). LC–MS-grade water, acetonitrile (ACN), chloroform, and methanol were obtained from Fisher Scientific. Antibodies for L-2HGDH and D-2HGDH were purchased from Proteintech Group Inc, Rosemont, IL, USA.

Animals: Mice were housed in a climate-controlled, light-regulated facility with a 12:12 h light–dark cycle with water and chow ad libitum. At 24 weeks of age, prior to sacrifice, blood and urine were collected. Plasma was obtained by centrifuging blood in ethylenediaminetetraacetic acid-coated tubes for 2 min at 5000× *g*. Before sacrifice, mice were fasted for 4 h. Kidney was perfused with ice-cold PBS through the left ventricle, and the kidney was dissected for the cortex region on ice. All samples were snap-frozen and stored at −80 °C until analysis. The study was conducted according to the guidelines of the University of Michigan Committee on Use and Care of Animals (project identification code: PRO00009416, date of approval: 14 February 2020).

Cell culture: Cells were grown in Dulbecco’s Modified Eagle Medium, low glucose (Thermo Fisher Scientific, Carlsbad, CA, USA), supplemented with 10% heat-inactivated fetal bovine serum (FBS; Corning^®^, Thermo Fisher Scientific, Carlsbad, CA, USA) and 1% penicillin/streptomycin (Thermo Fisher Scientific, Carlsbad, CA, USA), at 37 °C in 5% CO_2_. Cells were cultured between 1 and 10 passages. Media were changed every 24 h. For experiments, 4 × 10^5^ cells were plated in 6-well plates per well and grown to 80% confluence. Cells were serum-deprived for 24 h and cultured with 5 mM glucose (low glucose), 25 mM glucose (high glucose), and 5 mM glucose with 20 mM mannitol (osmotic control) supplemented with 0.1% FBS and 1% pen/strep for 48 h. The experiment was performed in triplicate, with two extra wells for each condition for cell counting in order to normalize metabolite concentrations. Cell counts were conducted with hemocytometers and 0.4% trypan-blue solution to assess for cell viability.

Creatinine analysis: Urine creatinine was measured, as previously published [47]. Plasma creatinine was measured with the same method by precipitating protein by pipetting 10 µL of plasma into 90 µL of methanol and injecting 5 µL for analysis.

2-HG (L- and D-Isomer) sample preparation and analysis: Samples were prepared using modified versions of previously reported [18]. Briefly, 0.5 mL of a chilled mixture of methanol, chloroform, and water (7:2:1) containing 1 uM of the internal standard, ^13^C_5_-L-2HG, was added to 15 µL of plasma or urine volume consisting of 200 pmols of creatinine. Tissue samples and HK-2 cells were homogenized and sonicated in methanol, and chloroform and water with 1 uM of the internal standard were added to a final ratio of 1:2:1 of methanol: chloroform: water. All samples were centrifuged at 17,000× *g* for 10 min at 4 °C, and the supernatant was transferred to a glass vial and dried under nitrogen. For tissue and cell culture samples, only the resulting upper layer was dried. Samples were then derivatized with DATAN in dichloromethane: acetic acid (4:1) solution at 75 °C for 1 h. Samples were dried in a speed-vac at 45 °C for 1 h. A standard curve of a mixture of D- and L-2-HG authentic samples was created and derivatized in the same manner as the samples for quantification and retention time confirmation purposes. Samples were reconstituted in water, and 5 µL were injected. LC-MS analysis was performed on an Agilent system consisting of a 1290 UPLC module coupled with a 6490 or 6495 triple quadrupole mass spectrometer (Agilent Technologies, Memphis, TN, USA). Samples were separated as previously reported [18]. Source conditions were as follows: gas temperature 200 °C, gas flow 14 L/min, nebulizer 35 psi, and capillary voltage 3000 V.

TCA cycle metabolite sample preparation and analysis: Kidney cortex samples were prepared as previously published [48]. Internal standards were added each at final concentrations of 20 uM during sample preparation. Samples were reconstituted in 2:1 ACN: water, and 5 µL was injected for analysis. A standard curve of a mixture of authentic samples of all measured metabolites was created and processed in the same manner as the samples for quantification and retention time confirmation purposes. LC-MS analysis was performed on an Agilent system consisting of a 1290 UPLC module coupled with a 6495 triple quadrupole mass spectrometer (Agilent Technologies, CA, USA). Metabolites were separated on an InfinityLab Poroshell120 HILIC-Z, 2.7 μm, 2.1 × 150 mm column (Agilent Technologies, Memphis, TN, USA). Mobile phase A was composed of 90% 10 mM ammonium acetate pH 9.0 with ammonia and 10% acetonitrile (ACN) with 2.5 uM InfinityLab Deactivator Additive (Agilent Technologies, Memphis, TN, USA); mobile phase B was composed of 15% 10 mM ammonium acetate pH 9.0 with ammonia and 85% acetonitrile with 2.5 uM InfinityLab Deactivator Additive. The flow rate was 0.25 mL/min, and the gradient was as follows: 0–2 min at 95% B, 2–5 min at 95% B, 5–5.5 min at 86%, 5.5–8.5 min at 86% B, 8.5–9 min at 84% B, 9–14 min at 84% B, 14–17 min at 80% B, 17–23 min at 60% B, 23–26 min at 60% B, 26–27 min at 95% B, and 27–35 min at 95% B. Column compartment temperature was kept at 25 °C. Data were acquired in negative mode. Transitions for compounds were as follows: α-ketoglutarate 145 → 101 *m*/*z*, _13_C^5^ α-ketoglutarate 150 → 105 *m*/*z,* cis-aconitate 173 → 85 *m*/*z*, citrate/isocitrate 191 → 110.9 *m*/*z*, _13_C^6^ citrate 191 → 110.9 *m*/*z*, fumarate 115 → 119 *m*/*z*, _13_C^4^ fumarate 119 → 74 *m*/*z*, malate 133 → 71.1 *m*/*z*, _13_C^4^ malate 133 → 71.1, *m*/*z*, succinate 117 → 72.8 *m*/*z*, _13_C^4^ succinate 121 → 76.1 *m*/*z*. Source conditions were as follows: gas temperature 225 °C, gas flow 13 L/min, nebulizer 35 psi, and capillary voltage 3500 V.

Western blot: Kidney cortex samples were homogenized in 2% SDS, 10% glycerol, and 0.05 mM Tris pH 6.8 with 1X Halt™ Protease Inhibitor Cocktail (Thermo Fisher, Carlsbad, CA, USA). The lysate was sonicated on ice for 30 s and centrifuged at 4 °C for 10 min at 17,000× *g*. The supernatant was quantified for protein concentration with the DC protein assay kit (Bio-Rad, Richmond, CA, USA). A total of 20 ug of protein was separated on 12% SDS-PAGE gel and transferred to PVDF membranes. Membranes were probed with D2HGH and L2HGH antibodies.

Data analysis and statistics: Quality control samples were made by pooling all the samples in the queue and run intermittently to control for machine drift and sample stability. Peak areas were extracted with Agilent Mass Hunter Workstation Software Quantitative Analysis for QQQ version B.07.01. Peak areas were normalized to internal standards before quantification. All statistics were performed with GraphPad Prism 7. Data were analyzed using student’s *t*-test with FDR correction where indicated or three-way ANOVA with Tukey’s post hoc correction.

## Figures and Tables

**Table 1:** Concentration of TCA cycle metabolites from *db*/*+* vs. *db*/*db* kidney cortex. Concentration of each metabolite was normalized to tissue protein. Q-value was calculated with Benjamini, Krieger, and Yekutieli procedure.

TCA Cycle Metabolite	Concentration *db*/*+* (SD)	Concentration *db*/*db* (SD)	*p*-Value	*q*-Value	Fold Change
α-ketoglutaric acid	37.0 (0.41) pmol/mg	88.9 (0.97) pmol/mg	0.011	0.0133	2.4
fumarate	0.98 (0.31) nmol/mg	1.67 (0.53) nmol/mg	0.008	0.0126	1.7
malate	1.63 (0.49) nmol/mg	3.27 (1.28) nmol/mg	0.005	0.0125	2.4
succinate	1.50 (0.63) nmol/mg	2.42 (0.43) nmol/mg	0.006	0.0125	1.6
cis-aconitate	94.5 (123) pmol/mg	747.5 (475) pmol/mg	0.002	0.0125	7.9
citrate/isocitrate	0.64 (0.24) nmol/mg	1.26 (0.56) nmol/mg	0.014	0.0142	1.9

## Data Availability

The data presented in this study are available in the article “Urinary 2-Hydroxyglutarate enantiomers are markedly elevated in a murine Model of Type 2 Diabetic Kidney Disease” and in the Supplementary Materials.

## Supplementary Materials

The following are available online at https://www.mdpi.com/article/10.3390/metabo11080469/s1, Table S1: TCA cycle metabolites in *db*/*db* and *db*/+ plasma, Table S2: TCA cycle metabolites in *db*/*db* and *db*/+ urine, Table S3: TCA cycle metabolites in HK-2 cells.
