# Supplementary material for: Urinary 2-Hydroxyglutarate Enantiomers Are Markedly Elevated in a Murine Model of Type 2 Diabetic Kidney Disease
Source: Metabolites. 2021 Jul 21;11(8):469. doi: 10.3390/metabo11080469 (PMC8400583; doi:10.3390/metabo11080469)
Supplement: Supplementary file 1 [file metabolites-11-00469-s001.zip › metabolites-1271918-supplementary.pdf]

Supplementary Materials:

Urinary 2-Hydroxyglutarate enantiomers are markedly elevated in a murine Model of Type 2 Diabetic Kidney Disease

Judy J. Baek [1], Subramaniam Pennathur [1,2*]

[1] Department of Molecular and Integrative Physiology, University of Michigan, Ann Arbor, MI 48105, USA; judybaek@umich.edu
[2] Division of Nephrology, Department of Internal Medicine, University of Michigan, Ann Arbor, MI 48105, USA; spennath@umich.edu
* Correspondence: spennath@umich.edu

TCA cycle metabolite - plasma	concentration *db/+* (SD)	concentration *db/db* (SD)	p-value	q-value
α-ketoglutaric acid	7.15 (5.97) pmol/µL	4.79 (1.82) pmol/µL	0.3051	0.6163
fumarate	2.15 (0.64) pmol/µL	3.61 (1.65) pmol/µL	0.0533	0.1615
malate	5.81(1.22) pmol/µL	9.94 (5.00) pmol/µL	0.0468	0.1615
succinate	2.71 (3.92) pmol/µL	4.52 (7.67) pmol/µL	0.5849	0.8861
cis-aconitate	0.68 (0.44) pmol/µL	0.69(0.41) pmol/µL	0.9936	1.0000
citrate/isocitrate	44.9 (13.9) pmol/µL	43.5 (9.03) pmol/µL	0.8213	0.9954

Supplemental Table S1: TCA cycle metabolites in *db/db* and *db/+* plasma. Concentration of each metabolite was normalized to plasma volume. Q-value was calculated with Benjamini, Krieger and Yekutieli procedure. *n* = 7-8. 20 µL of plasma and 10 µL of 10 µM of TCA cycle metabolite internal standards in water were added to 120 uL of ice-cold methanol. The resulting solution was centrifuged at 17,000 x g for 10 min and the supernatant was dried under nitrogen. Samples were reconstituted in 30 µL of 2:1 acetonitrile: water solvent and samples were analyzed as described in the methods section.

TCA cycle metabolite - urine	concentration *db/+* (SD)	concentration *db/db* (SD)	p-value	q-value
α-ketoglutaric acid	30.8 (15.8) mmol/mol	54.8 (24.9) mmol/mol	0.0472	0.2439
fumarate	0.59 (0.08) mmol/mol	0.77 (0.44) mmol/mol	0.4859	0.4908
malate	4.87 (1.50) mmol/mol	3.31 (5.57) mmol/mol	0.3118	0.4908
succinate	7.68 (3.90) mmol/mol	4.61 (2.26) mmol/mol	0.0805	0.2439
cis-aconitate	6.28 (1.53) mmol/mol	5.56 (2.23) mmol/mol	0.4841	0.4908
citrate/isocitrate	182 (29.2) mmol/mol	203 (73.6) mmol/mol	0.4784	0.4908

Supplemental Table S2: TCA cycle metabolites in *db/db* and *db/+* urine. Concentration of each metabolite was normalized to creatinine. Q-value was calculated with Benjamini, Krieger and Yekutieli procedure. *n* = 7 – 8. Urine volume equating to 200 nmol of creatinine and 30 µL of

100 µM of TCA internal standards in water were dried under nitrogen. Samples were reconstituted in 300 µL of 2:1 acetonitrile: water and samples were analyzed as described in the methods section.

**TCA cycle metabolite - cells**	**Low Glucose (SD) pmol/10^5 cells**	**High Glucose (SD) pmol/10^5 cells**	**Osmotic Control (SD) pmol/10^5 cells**
α-ketoglutaric acid *†§	6.18 (0.37)	7.96 (0.55)	4.31 (0.55)
fumarate *†§	15.4 (1.40)	17.7 (1.34)	10.9 (0.52)
malate *†§	31.7 (1.54)	35.2 (1.37)	24.8 (1.50)
succinate *†§	6.14 (1.09)	13.9 (0.97)	10.3 (0.81)
cis-aconitate *†	0.49 (0.05)	0.79 (0.23)	0.45 (0.09)
citrate/isocitrate *†	12.5 (1.31)	16.3 (1.09)	11.3 (0.40)

Supplemental Table S3: TCA cycle metabolites in HK-2 cells under 5 mM glucose (low glucose), 25 mM glucose (high glucose), and 5 mM glucose and 20 mM mannitol (osmotic control) Concentration of each metabolite was normalized to the cell count. * : adjusted p-value < 0.05 for low glucose vs high glucose, †: adjusted p-value < 0.05 for osmotic control vs. high glucose, §: adjusted p-value < 0.05 for low glucose vs. osmotic control. One-way ANOVA with Tukey's post-hoc correction. *n* = 4.
